# Enabling relational leadership in primary healthcare settings: lessons from the DIALHS collaboration

**DOI:** 10.1093/heapol/czx135

**Published:** 2018-07-08

**Authors:** Susan Cleary, Alison du Toit, Vera Scott, Lucy Gilson

**Affiliations:** 1 Health Economics Unit, School of Public Health and Family Medicine, University of Cape Town, Cape Town, South Africa; 2 Self Employed Industrial and Organizational Psychologist and Time to Think coach, Cape Town, South Africa; 3 School of Public Health, University of the Western Cape, Cape Town, South Africa; 4 Health Policy and Systems Division, School of Public Health and Family Medicine, University of Cape Town, Cape Town, South Africa and; 5 Department of Global Health and Development, London School of Hygiene and Tropical Medicine, London, UK

**Keywords:** Leadership development, governance and accountability, primary healthcare

## Abstract

Strong management and leadership competencies have been identified as critical in enhancing health system performance. While the need for strong health system leadership has been raised, an important undertaking for health policy and systems researchers is to generate lessons about how to support leadership development (LD), particularly within the crisis-prone, resource poor contexts that are characteristic of Low- and Middle-Income health systems. As part of the broader DIALHS (District Innovation and Action Learning for Health Systems Development) collaboration, this article reflects on 5 years of action learning and engagement around leadership and LD within primary healthcare (PHC) services. Working in one sub-district in Cape Town, we co-created LD processes with managers from nine PHC facilities and with the six members of the sub-district management team. Within this article, we seek to provide insights into how leadership is currently practiced and to highlight lessons about whether and how our approach to LD enabled a strengthening of leadership within this setting. Findings suggest that the sub-district is located within a hierarchical governance context, with performance monitored through the use of multiple accountability mechanisms including standard operating procedures, facility audits and target setting processes. This context presents an important constraint to the development of a more distributed, relational leadership. While our data suggest that gains in leadership were emerging, our experience is of a system struggling to shift from a hierarchical to a more relational understanding of how to enable improvements in performance, and to implement these changes in practice.

**Key Messages**

- Relational leadership—associated with practices such as mentoring/coaching others, and enabling the relationships and commitment needed to work together to achieve common purpose—has been identified as important in strengthening the performance of health systems.
- In LMICs, relational leadership can be strengthened through collaborative reflective practice that pays attention to values and relationships.
- In the experience reported here, gains from relational LD included increased trust and team cohesion across and within levels of the district health system.
- The strongly task-oriented governance context with multiple bureaucratic accountability mechanisms was however perceived to constrain relational LD.

## Introduction

Strong management and leadership competencies have been identified as critical to strong health systems (Egger *et al.* 2005; de Savigny and Adam 2009). While this is true in all health systems, the resource scarcities and crises (Elloker *et al.* 2012) of Low- and Middle-Income Country (LMIC) settings make it all the more important that leadership supports health workers to do their best to deliver the quality, responsive care afforded by the available resources (Daire *et al.* 2014). A recent report from the Alliance for Health Policy and Systems Research (2016) argues that literature on leadership from LMICs is sparse, and much of what is available focuses on individual leaders. Instead, as the report argues, ‘it is the sturdiness of the system as a whole, with leadership exercised effectively at multiple levels, which will stand the test of a serious challenge. In health, this calls for a harmonious confluence of leadership across a wide array of actors, to collectively create a strong leadership for the health system.’ (p. 15)

While leadership literature from LMICs is limited, a body of work does however point to the importance of relationships in health systems strengthening. For example, the growing literature on people-centred health systems understands health system strengthening to be about engaging the people, relationships, norms and values of the health system, to implement actions to strengthen it (Sheikh *et al.* 2014). In Ghanaian hospitals, findings suggest that a balanced combination of task-oriented approaches with commitment-eliciting human resource practices—such as perceived supervisor support—can induce a climate characterized by organizational commitment and trust (Marchal *et al.* 2010; Marchal 2011). In South Africa, a case study of the leadership roles of primary care facility managers (FMs) suggests that managing people and relationships, and self in relation to others, is the primary demand of daily practice (Daire and Gilson 2014). Finally, in a case study of priority setting practices in two Kenyan hospitals, in one hospital, the medical superintendent managed relationships and balanced power differences in ways that enabled the perception of a fair priority setting process, while in the other hospital, with a different management style, the process was perceived to be unfair, with negative implications for morale (Barasa *et al.* 2016).

Although still unusual in LMIC debates, these ideas regarding the importance of relationships in health systems strengthening are mirrored within the broader high income country leadership literature. In this literature, the notion of ‘relational leadership’ positions leadership as an interpersonal phenomenon associated with collaboration, empathy, trust and empowerment (Cummings *et al.* 2010); as non-hierarchical and distributed; and embedded within everyday interactions, conversations and relational processes (Cunliffe and Eriksen 2011). In this conceptualisation, leadership is not restricted to those holding managerial positions, but rather is a process of mutual influence through which an emerging social order and/or actions are co-constructed (Uhl-bien 2006).

Given the potential importance of relationships and the promise of a distributed relational leadership for strengthening health systems, it is important for health policy and systems researchers to build the LMIC evidence base of the outcomes of such leadership and how it might be nurtured. However, in learning lessons about how to enable leadership development (LD), a number of evaluation challenges need to be borne in mind. Firstly, there are a variety of LD approaches, including group or individual coaching, mentoring, reflection, action learning, 360-degree feedback, job assignments and community of practice approaches, amongst others (Day 2000; Ely *et al.* 2010; Marquardt *et al.* 2010; Carey *et al.* 2011; Cummings *et al.* 2013; Daire *et al.* 2014; Mcnamara *et al.* 2014; Doherty and Gilson 2015; Leggat *et al.* 2015). Secondly, as argued by Ely *et al.* (2010), an LD intervention needs to be tailored to the particular context—and even within similar organizational settings, the eventual LD design would need to contain different LD approaches, addressing different (groups of) participants and implemented at different times. Thus, while experimental study designs can provide insights into *whether* one particular approach to LD is effective, it is also important to generate insights into *how* the interventions function and have impact (Ely *et al.* 2010).

As part of the broader DIALHS (District Innovation and Action Learning for Health Systems Development) collaboration, this article reflects on 5 years of action learning and engagement around leadership and LD within primary healthcare (PHC) services in a relatively resource-poor South African setting. Working in one sub-district in Cape Town, we co-created LD processes with FMs from nine PHC clinics and with the six members of the sub-district management team (SDMT). Within this article, we describe the overarching LD design that emerged through our collaboration, explain the governance context in which it was implemented and consider whether and how our approach to LD has, so far, enabled relational leadership.

## Methods

### Setting and the DIALHS project

The setting for this study is the Mitchell’s Plain sub-district within the City of Cape Town in the Western Cape Province of South Africa, a relatively low income community of approximately 500 000 people with a high burden of disease including Human Immunodeficiency Virus, Tuberculosis, chronic non-communicable disease, gang-related violence and road traffic accidents (Klipfontein–Mitchell's Plain Orientation Guide 2013). Within this setting, public health services are provided by the provincial government of the Western Cape and by the City of Cape Town, a local government authority.

Since 2010, researchers from two universities have worked with managerial colleagues from both authorities in a long-term collaborative project (DIALHS). Amongst other aims, the project seeks to understand leadership and management in the sub-district, and how it is influenced by the district, provincial and national structures, processes and policy environment in which it is located. In this study, we are also mindful of the influence of the local context, and the challenge this poses for LD:

In Tafelsig [clinic] they [stole] the palisade [perimeter] fencing twice … very frustrating to manage these things – we sit and just manage repairs. The violence! There were gunshots this morning … It’s a huge thing…We are losing so much and it’s frustrating … It’s difficult to keep your cool” (SDMT reflective meeting, December 2014). Within such a context, the reality of LD interventions is that “all are working under extremely stressful conditions all the time, the entire system is stressed, which then favours a compliance mindset [of simply following instructions]. And yet, under stress and in a compliance and punitive environment, FMs and everybody else are less likely to listen and the ability to change is reduced (LD/research team reflective meeting, May 2014).

The overall project approach was one of collaborative action learning—‘a collective process for inquiring into and taking action on projects and practices within their complex, multi-agent contexts’ (Rigg 2011, p. 15). We have understood reflective practice as a means to learn from experience (Schon 1983; Mann *et al.* 2009) and have also recognized that the ‘formation of communicative space’ is a form of action (Kemmis 2001, in Reason, 2006, p. 193). In other words, the learning is itself an intervention in the system.

**Figure 1.**
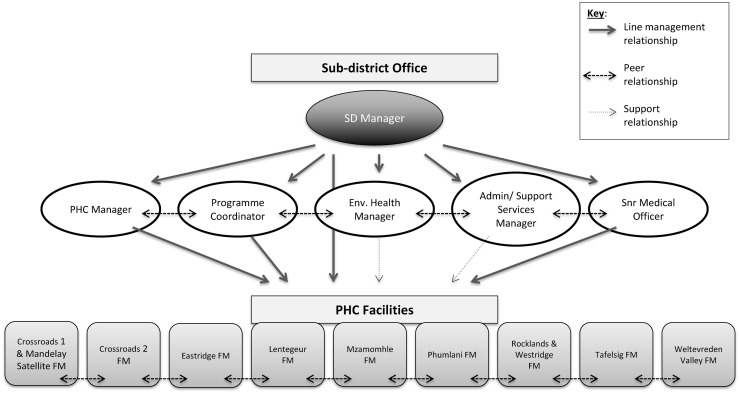
Actors, settings and relationships framing the LD interventions (as of January 2016)

The LD initiatives that are the subject of this paper were developed with City of Cape Town authorities only. Figure 1 depicts the health system actors, organizational settings and managerial relationships that have framed the engagements that are the subject of this article. The LD/research team that was involved in the leadership engagements included an organizational psychologist and a number of health policy and systems researchers. While the LD/research team regularly engaged as a group in order to support learning and reflexivity, in general terms, the organizational psychologist took the lead in designing and implementing the LD interventions, while the researchers took the lead in making meaning from the experiences and were less involved in the implementation of the LD interventions. The LD interventions were deliberately framed by *Thinking Environment* principles, explained further later, in which the organisational psychologist (AdT) and one member of the research team (SC) are trained.

### Research approach

Within the umbrella of the action learning design of DIAHLS, the research approach was flexible and qualitative. The LD intervention emerged through a series of engagements undertaken over time, as described in more detail later, which were accompanied by regular reflective discussions among the research team and with managerial colleagues, all of which were documented. In considering whether and how the development of a distributed relational leadership was enabled, we also draw on document reviews, in-depth interviews, group discussions, observations of practice and of LD interventions, and, again, the notes from the regular reflective discussions. Our data for this article therefore include observational and interview notes, transcripts from reflective discussions, and written reports from the organizational psychologist. We also draw insights from additional research conducted by an external evaluator (Podems Consulting 2014). A summary of these data is presented in Table 1. The monthly half-day research team meetings held throughout the project combined with regular reflective discussions with the SDMT and PHC FMs, enhance the rigour of our analyses, by supporting reflexivity, and allowing emerging insights to be tested. Ethical approval was granted by the University of Cape Town Research Ethics Committee and permission for the work was obtained from the City of Cape Town. More details on the overall approach and methodology are available in a published source (Lehmann and Gilson 2015).

**Table 1.** Summary of data

Type of data	Timing	Details
In-depth interviews and discussions	2011–2016	12 interviews
Reflective discussions within research and LD team	2011–2016	Monthly reflective discussions (typically half day meetings)
Reflective discussions including sub-district colleagues	2012–2016	7 reflective discussions (typically half to full day meetings)
Observations of LD processes	2013–2016	15 group coaching processes and 1 relational leadership skills workshop observed; teaching on 5 day health management course
Report on FM coaching (Barak and du Toit 2013)	2012–2013	1 report
Observations of FM peer support process	2013–2015	5 meetings observed
Observations of facility supervision visit process	2014	4 days’ facility supervision and 2 feedback sessions observed
Report from external evaluator (Podems Consulting 2014)	2015	1 report, including insights from 13 interviews

To make meaning from these experiences for this article specifically (Patton 1999; Barnett-Page and Thomas 2009), the first author read through the observational, interview and reflective discussion data as well as the documents, reports and publications from the overall DIALHS project, and extracted all data elements that spoke about management, leadership, the governance context of the sub-district and the LD interventions. These data were then summarized chronologically and according to type within a document that formed the basis for a reflective discussion within the LD/research team. In addition, the first author and the organizational psychologist engaged in three additional reflective discussions. Thereafter, the first author used thematic analysis to interpret the data using themes generated both from relevant bodies of literature and from the data themselves, and the initial analysis was then discussed within the author team. This process led to the development of a draft article, which was presented to the SDMT within a further reflective discussion as well as twice reviewed by the writing team.

## Results

### The emergent LD interventions

As the specific LD interventions were nested within the overall action learning design of the DIALHS collaboration, they intentionally emerged from processes of reflective learning with health system managers and were rooted in everyday sub-district realities. Within this emergent design, we drew structure from the *Thinking Environment* as a methodology that is appropriate for enabling a distributed relational leadership. The *Thinking Environment* is underpinned by a number of perspectives, including: (1) the quality of everything we do depends on the quality of the thinking we do first; (2) the quality of our thinking depends on the quality of our relationships; (3) the mind that holds the question or issue can most *effectively* resolve it; and (4) while human nature cannot be proven to be inherently good or bad, explicitly assuming a positive view on human nature (e.g. intelligent, resilient, adaptive, innovative, caring) will more effectively enable high-quality thinking and relating than if we were to assume a more negative perspective (Kline 2008). In essence, the first two perspectives emphasize the importance of relationships for productive team engagements and learning, the third emphasises the importance of allowing colleagues to find the solutions to their own challenges and the fourth emphasises the importance of trust for interpersonal engagements.

In addition to these four perspectives, the *Thinking Environment* rests on 10 components—behaviours, attitudes, values and beliefs—that together are argued to create the culture and relationships needed to enable productive team engagements. Table 2 summarizes these 10 components, with illustrative examples of relationships and values in local health system governance drawn from the action learning insights. In essence, we drew on *Thinking Environment* understandings and approaches in the way that we facilitated the processes of reflection, group coaching and relational leadership skills building that emerged within the LD initiatives.

**Table 2.** Ten components of the *Thinking Environment* related to health system governance, relationships and values

Thinking environment component	Description	Implications for health system governance, relationships and values
Attention	Listening with palpable respect and interest, and without interruption	Reinforce the culture of paying respectful attention to colleagues and patients within all engagements (e.g. meetings, performance appraisals, one-on-ones, patient consultations)
Equality	Treating each other as thinking equals	Despite rank and hierarchy, seek to amplify points of equality within engagements (e.g. all perspectives are useful, all have potential to think well)
Ease	Offering freedom from internal rush or urgency	Acknowledge the importance of employee wellness for performance and quality of care
Appreciation	Offering genuine acknowledgement of a person’s qualities. Practicing a 5:1 ratio of appreciation to criticism	Intentionally seek to identify good practice; minimize the potential for demotivation from the implementation of accountability mechanisms that focus on compliance
Encouragement	Giving courage to go to the cutting edge of ideas by moving beyond internal competition	Use encouragement to create sufficient psychological safety for teams to problem solve, adapt and innovate
Feelings	Allowing sufficient emotional release to restore thinking	Acknowledge that empathising with the concerns and frustrations of colleagues is an important part of leadership practice, particularly in stressful, under-resourced working environments
Information	Supplying the facts	Develop the capacity to provide information in a way that promotes understanding (e.g. implementing new policies while taking cognisance of sub-district constraints and realities). Value the experiential knowledge of managers
Diversity	Welcoming divergent thinking and diverse group identities	Respect the diverse cultures and identities of colleagues and patients.
Incisive questions	Removing assumptions that limit independent, creative thinking	While acknowledging the truth, or potential truth, of the assertion (e.g. the budgets are inadequate) seek to craft a question or focus that can move thinking forward (e.g. given the budgets that we receive, what package of quality services can we provide to the community?)
Place	Creating a physical space that says to people ‘you matter’	Consider how health facilities (patient waiting areas, toilets, staff room, offices) and administrative building spaces can better demonstrate respect and care, for example by removing broken furniture and equipment

**Figure 2.**
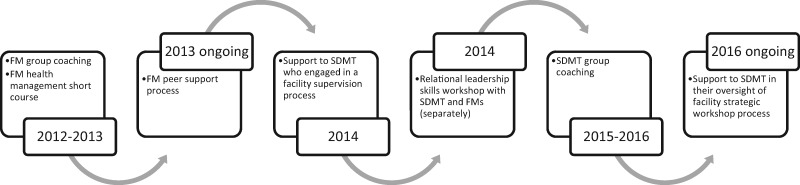
Cycles of LD interventions

The LD interventions that emerged through this process of reflection and action are illustrated in Figure 2, and are described in more detail in Table 3. Starting from 2012, the first intervention to be implemented was a process of seven FM group coaching sessions (2012–2013). Thereafter, in 2013, six FMs were supported to attend short course training in health management, accompanied by some ‘light touch’ follow-up engagement with the LD/research team. These activities were intended to broaden FM’s understandings of management and to support continued peer-to-peer engagement. As a continuation of these engagements, the FM group initiated their own process of peer support (2013 ongoing). Their intention was to have monthly half-day meetings where they could learn from each other and share best practice.

**Table 3.** Details of LD interventions

Intervention component	Description	Audience	LD/research team role(s)
*2012–2013* FM group coaching	Seven 2-h long sessions aimed at creating a community of practice. Included relational leadership skills building (e.g. enabling a *Thinking Environment* in the workplace, managing difficult conversations, etc.)	FMs	Facilitation of 7 group coaching sessions; writing of a report on the experience (Barak and du Toit 2013), 1 reflective workshop with SDMT to learn from experience
*2013* FM short course training in health management	Six FMs attended 5-day short course	FMs	Teaching role
*2013 to date* FM peer support	Monthly half-day meetings of FMs Intention to enable skills transfer and sharing of best practices within FM team	FMs	Interviews with 3 FMs; observations and light-touch facilitation of 5 peer support meeting
*2014* Facility supervision	Day-long supervision visits to each facility run by SDMT every six months. Intention to enable enhanced facility performance. Follow up feedback session, originally to FM and later to entire facility staff	District health system (SDMT, FMs, Facility Staff)	Observation of 4 supervision visits and 2 feedback sessions; 4 individual interviews; 1 reflective workshop with SDMT; and 1 joint SDMT-FM reflection to learn from experience
*2014* Relational leadership skills	Day-long workshop on how to enable a *Thinking Environment* in the workplace with a particular focus on productive practices and cultures for small/large group engagements (e.g. meetings, team engagements, one-on-ones, etc.)	FMs, SDMT	Facilitation of 1 relational leadership skills workshop; 4 individual interviews; 1 reflective workshop with FMs, and 1 reflective workshop with SDMT to learn from experience
*2015–2016* SDMT group coaching	Eight 2-h long sessions aimed at creating a community of practice. Included relational leadership skills building (e.g. enabling a *Thinking Environment* in the workplace, understanding own leadership identity)	SDMT	Facilitation of 8 group coaching sessions; observations of each coaching session; 1 reflective workshop with SDMT to learn from experience
*2016* Facility strategic workshops	Day-long strategic planning workshops in each facility. Initiated and supported by SDMT, but facilitated by FMs	Whole system	Facilitation of reflective conversations thinking through the SDMT role in the sessions (as part of group coaching); provision of specific skills inputs

Then when, in early 2014, the SDMT signalled their intention to change the way in which they were conducting their facility supervision visits and asked for support from the LD/research team, this provided an opportunity to take the lessons from the previous FM support processes forward. While plans for the new supervisory visits evolved over time, the overall intention from the perspective of SDMT was for the entire team to visit each facility for a full day, and to implement a process of ‘support and mentorship through monitoring and evaluation’. The LD/research team sought, in turn, to observe these processes and to engage in reflective conversations with the SDMT and FMs so as to enable them to better achieve their intentions. During this process of supporting change in facility supervision practice, a need was identified for specific leadership skills. This was addressed through the provision of a day-long workshop to the SDMT and FM groups with particular emphasis on how to facilitate generative team engagements, meetings and one-on-ones.

The final LD intervention that is considered in this article is a process of eight SDMT group coaching sessions (2015–2016). Again, the coaching was embedded within the *Thinking Environment* methodology and the overall approach within each session involved working with the current governance and leadership concerns that were raised by the SDMT. During the group coaching, the SDMT reflected specifically on their intention to initiate, and then their experience of running, a process they deliberately termed ‘facility strategic planning’ as this term aligned with usual organizational practice. For the SDMT this process entailed supporting each FM to facilitate a day-long session with their staff, in which the FMs used *Thinking Environment* principles and practices to enable all staff to discuss their work activities and experiences and identify their challenges and needs. In this process, the SDMT sought specifically to draw on their group coaching experience in the way they supported FMs and encouraged engagement with facility staff.

In summary, the *Thinking Environment* provided the base methodology for engagements, and engagements emerged through action-learning processes. In addition, within these processes, the LD initiatives allowed participants to deepen their understanding of identities (self) and their relationships, as explained further in Box 1.

Box 1: Engaging with understandings of self and relationships through LD processes

The LD processes **understood the self as the start of relational leadership.** They stressed the ‘importance of having a vision, a purpose and hence identity’ linked to the notion that ‘if I am clear about my identity and purpose then I can reflect on who I am being as a leader’ (Notes from FM coaching, July 2012). However, in this context positive identities lived alongside a sense of victimhood—a perception that the system ‘sees us as toilet paper’ or as ‘labourers’ (Notes from FM coaching, August 2012). The LD processes therefore aimed to ‘gain traction with increasing a sense of locus of control—shifting from a sense of blame (e.g. FMs feeling that staff aren’t committed enough) to a sense that—through using leadership practices—shifts can be achieved in staff and individual empowerment’ (LD/research team reflective meeting, September 2012). **Building on this foundation of personal LD**, the LD approach further sought to “make the benefit of relationships more visible so that the FM does not feel derailed from the ‘real’ tasks by managing relationships” (LD/research team reflective meeting, September 2012). The processes specifically aimed to enable FMs to develop the relationships that generate effective team engagements, and to build their capacity to trust their own insights in order “to develop their own [managerial] ‘how to’ kit’” (SDMT reflective meeting, July 2013). The LD processes also worked to promote a coherent understanding of individual and collective identity within the sub-district—recognizing that the ‘huge sense of service’ of health system actors, and the understanding that ‘work is passion and calling’ (Barak and du Toit, 2013, p. 4) provided an important platform for LD activities.

### The wider governance context

In order to consider whether and how these LD interventions influenced leadership, it is important to situate the analysis within context. In South Africa, while many managerial functions have been decentralized to the sub-district and facility level, accountability to city, provincial and national managers is maintained through the use of a number of bureaucratic mechanisms. For example, routine health information is captured by clerks within primary care facilities and used to inform planning, target setting and monitoring processes at the local government and/or provincial levels. The ‘National Core Standards’ process (National Department of Health 2011) is an example of a national accountability imperative involving lengthy checklists of facility buildings, equipment, structures and processes. Facility and sub-district managers are responsible for ensuring that these standards are met, and that procedures are implemented to address concerns arising from national complaints procedures, patient satisfaction surveys and clinical audits, amongst others (Scott *et al.* 2015).

This broader health system governance context was found to influence the leadership practices of the sub-district (Scott and Gilson 2017). For example, while the intention of the SDMT was to be supportive during their facility supervision visits, the imperatives derived from central accountability mechanisms meant that support and mentoring was dominated by a compliance-driven approach: ‘It’s supposed to be about mentoring and support—but is in fact a very detailed audit… The part of management that is very well looked at during the visit is that part that looks at whether the equipment is there, are people where they should be, are staff there on time … but not the side of management that looks at people.’ (LD/research team reflective meeting, April 2014). Similarly the LD/research team observed ‘a gap between what their [the SDMT] intention is (i.e. they have a stated intention of being supportive, and being useful and being generative)—which is a transformative way of engaging with their system—and how it manifests—they conduct the observations in a transactional way… The entry point for engaging becomes compliance.’ (LD/research team reflective meeting, April 2014). When interviewed, FMs similarly understood that the purpose of the supervisory visits was for ‘checking up—have systems been implemented or not’ (FM1 interview, April 2014) or ‘to see whether systems were in place, how they were working and to standardize them across all facilities according to policy’ (FM2 interview, April 2014).

In contrast, FMs expressed a preference for an alternative style of supervision, with ‘support and mentoring 70–80% of the time and monitoring and evaluation 20–30% of the time’ (SDMT-FM reflective meeting, June 2014). The FMs also commented that ‘their judgment was not always trusted by management. They felt that management ought to acknowledge their understanding of the reality of their local clinic context a bit more … and give them more leeway to make their own decisions based on their understanding of this local context’ (SDMT-FM reflective meeting, June 2014). Similarly, when interviewed about the supervisory visits, an FM commented: ‘Whatever feedback you give, whether positive or negative, it’s the way you give it that matters the most’ (FM1 interview, April 2014). When the research team brought this feedback to the SDMT, they acknowledged that although monitoring progress against targets was a critical part of their jobs, one should not ‘shoot the person when things are not working; rather sit down and think what other possibilities and options are. It’s important how you say something, not just what’ (SDMT-FM reflective meeting, June 2014).

Overall, therefore, despite an openness and desire for more supportive mentoring on the part of both the SDMT and FMs, and signs of a more relational leadership approach in the SDMT’s own practice (Gilson *et al.* 2014), the broader governance context and the need to comply with central accountability processes was judged to limit the impact of relational LD.

### Enabling relational leadership

Despite this broader governance context, the SDMT and FMs began to report changes in their understanding of the benefits of relational leadership: ‘We realized that our relationships were not good—all of them not good—we were all chasing numbers and statistics. The morale was poor and we were all burnt out. How could we do that—to our staff and ourselves?’ (SDMT1 reflection, October 2014). These shifts in understanding enabled a larger space for FMs to exercise discretion: “Before you were told that you must do ‘this, this and this’ and even though you have planned for your facility, you could not do your own things. [Now the SDMT] give me more space to do the things that I have prioritized.” (FM reflective meeting, November 2014). In addition, FMs reported improvements in the way in which engagements around the accountability processes were handled: ‘I used to be scared of these [accountability] meetings…they were used for venting. Now, they are more relaxed and cheerful and people are able to speak their mind in a positive way. There is not so much venting… They are much, much better’ (FM3 interview, October 2014). Within the context of meetings run using *Thinking Environment* principles, the use of positive rounds—an opportunity for each participant explicitly to verbalize their appreciation for the group in turn, or receive appreciation from another participant in a structured manner—was highly valued. As well as motivating managers, it was seen to lay a relational foundation that could then also support their ability to receive and work with constructive criticism (SDMT reflective meeting, October 2016).

Overall, the SDMT and FMs were positive about their exposure to the set of LD processes: “[DIALHS] can be a programme that is adopted by the health department because it can empower and retain people. I was ‘born again’ from a session with [the organizational psychologist]—she really changed me so much in that one mentoring session…Just by listening and being calm…It brings with it a certain culture.” (SDMT reflective meeting, December 2014). They also reported benefits from their use of the leadership skills: ‘last week was the third staff meeting where I’ve used [the new skills]…I noticed that in the main body of the meeting people were now talking as opposed to before when they would just sit and nod but make no input. The last meeting was nice, it was really very nice. They contributed a lot’ (FM4 interview, October 2014). FMs also mentioned that the ‘sub-district team has really improved in terms of support and feedback’ (FM5 interview, August 2015). Similarly, a sub-district manager mentioned that the strategic planning workshops facilitated by the FMs were ‘exceptional’; ‘[it was] such a nice experience to watch how dynamic they were. A gift to see all of this work come together’ (SDMT coaching notes, June 2016).

From the perspective of the SDMT (SDMT reflective meeting, October 2016), moreover, the health system gains attributed to the LD interventions included greater trust and cohesion within the SDMT and in the relationship with FMs and staff. This was characterized as being a shift in the organizational culture within the sub-district. The SDMT noted that, in their experience, the FMs were becoming more engaged and assertive; for example, they were beginning to speak up in meetings, to the extent that they could express their concerns about new initiatives when they thought them not feasible to implement. Previously they would have resorted to grudging compliance and later complained about the decisions. Finally, there were perceptions of, unconfirmed, broader performance gains, including reductions in patient complaints linked to staff attitudes—‘we receive more compliments these days than complaints’ (SDMT1 interview, October 2014), as well as reductions in ‘staff grievance cases’ (SDMT1 interview, November 2015).

## Discussion

This article reflects on 5 years of LD experience in a low-income setting within Cape Town, South Africa. In presenting our analysis, we build from the broader findings of our action learning collaboration, including: (1) the centrality of relationships between actors—for improving sub-district performance (Elloker *et al.* 2012; Scott *et al.* 2014), for community responsiveness (Cleary *et al.* 2015) and for the implementation of action learning processes (Lehmann and Gilson 2015); and (2) the importance of deepening understandings of identity and purpose both at the individual level, where transitioning from a clinical to a managerial identity is important for FM functioning (Daire and Gilson 2014), and at the collective level, where the strengthening of the PHC approach requires the nurturing of PHC-aligned values and mindsets (Gilson *et al.* 2014).

Our findings suggest that the sub-district is located within a central governance context that is hierarchical, with performance monitored through the use of multiple accountability mechanisms including standard operating procedures, facility audits and target setting processes. While accountability mechanisms can be important for enhancing health system functioning, the way in which these mechanisms are implemented can have an impact on whether they are motivating or demotivating to health system actors, and on whether they generate a more hierarchical or a more relational culture (Cleary *et al.* 2013). Hupe and Hill (2007) relate this to different modes of governance which create different relationships and values. The enforcement mode is associated with compliance to rules and to standard operating procedures, while the co-production mode is associated with the commitment to shared purpose (Hupe and Hill 2007).

Our early experience of the manner in which the SDMT responded to the central governance imperatives was that enforcement was the dominant mode of implementing accountability mechanisms in the sub-district, with negative implications for morale. We also judged that the dominance of these mechanisms crowded out the space for more relational approaches. Indeed, in linked DIALHS research, fear of missing targets has been cited as one reason why FMs become over-involved in providing clinical services, limiting the available time for their leadership responsibilities (Daire and Gilson 2014). However, in keeping with others who argue that leadership styles do not entail an ‘either/or’ dichotomy (Cummings *et al.* 2010; Marchal *et al.* 2010, 2011) our findings suggest that relational processes can complement bureaucratic accountability processes. For example, the review of targets can be done in a manner that is respectful of relationships towards the co-production of better health service delivery (for a fuller DIALHS analysis of this, see Scott and Gilson 2017).

Our approach to LD has a number of key characteristics. Firstly, we used the *Thinking Environment* (Kline 2008) as our base methodology in conducting our support for managers. Amongst other benefits, this methodology enables the trusting relationships (Carey *et al.* 2011) and safe environment (Edmondson 2003; Nembhard and Edmondson 2006) that literature suggests is needed for LD. Through the use of the ten components (Table 2), the *Thinking Environment* approach also allows for explicit norms for interpersonal engagement to be set. Based on a review of the group development literature, it is argued that explicit norms are critical for team success in that they can encourage respectful communication, distribute power to the weaker members of the group, and generate feelings of trust and belonging which in turn enhances the commitment of team members to learning together (Marquardt *et al.* 2010). Secondly, we sought to work with the key governance and leadership challenges and opportunities that were raised by the SDMT and FMs. This is argued to be an essential LD approach in the literature (Day 2000; Day *et al.* 2014) and reflects the coaching principle of allowing for the questions and answers to rest with the client (Byrne 2007; Ammentorp *et al.* 2013). Our approach also enabled us to role model the value of trusting in the abilities of others to find solutions to their own challenges. Thirdly, the implementation of each LD intervention was followed by at least one reflective discussion between research and practitioner partners in order to learn from experience, also allowing the leadership capability of reflection to be nurtured (Horton-Deutsch and Sherwood 2008). And finally, our LD processes were all group or team based (e.g. group reflection or group coaching) and worked across two different layers of the system, allowing us to promote a distributed leadership within the sub-district as opposed to focusing on individual leader capabilities (Day 2000; Atwood *et al.* 2010; Day *et al.* 2014).

While our study was not designed to measure impact in a quantitative sense, qualitative insights suggest an emerging shift towards a more relational leadership, reflected in the actual ability to strengthen supportive relationships. These shifts include enhanced trust in the ability of FMs to use their discretion to improve services in their facilities, greater respect in the way that accountability processes were managed, and enhanced mentoring skills, with hints of the potential for broader performance gains linked to reduced patient complaints and staff grievance cases. Nonetheless, our experience suggests that the broader health system governance context might continue to be an important constraint to distributed LD.

Although our study is, to the best of our knowledge, one of the first to report on efforts to strengthen relational leadership in an LMIC health system, these findings are not unique; the transformation of health system cultures is challenging in many settings. For example, evaluations from a long-term LD programme within the Alberta Cancer Board in Canada showed mixed results (Sharlow *et al.* 2009; Cummings *et al.* 2013). In qualitative work, findings suggested that participants felt that individual change was impossible without organizational change (Spiers *et al.* 2010). Several studies within the UK’s National Health Service (NHS) have shown similar findings. In a study of reflective practice, the authors argued that reflection needs to be a legitimated organizational process in order for it to become an opportunity for organizational transformation (Nicolini *et al.* 2003) while a study of distributed change leadership suggested that the presence of distracting strategic priorities and fragmented organizational structures was found to impede progress (Fitzgerald *et al.* 2013). A third NHS study into the implementation of strategic human resource management found, moreover, that ‘staff were mostly keen, creative, comfortable with new ways of working if these improved patient care, and hungry for training and development that would help them do their jobs better. But structural barriers at both national and local level were multiple and pervasive, and they accounted for more delayed or diverted initiatives than any other single factor in our overall analysis’ (Macfarlane *et al.* 2011, p. 66). More optimistically, an ethnographic study into the implementation of new regulations regarding working hours for surgical residents in two American teaching hospitals suggests that the creation of relational spaces—‘areas of isolation, interaction and inclusion that allow middle-manager reformers and subordinate employees to develop a cross-position collective for change’ (p. 657) can help those in favour of the proposed change to come together in order to challenge resistance (Kellogg 2009).

There are a number of implications of this study for those who wish to influence policy and practice in the space of health leadership and LD in LMICs. Firstly, the development of distributed relational leadership has value, and is in line with a recent call by the Alliance for Health Policy and Systems Research (2016) for the development of participatory leadership. Secondly, national senior policymakers need to be exposed to evidence regarding the value of relational leadership for health systems strengthening, as well as on the ways in which centrally driven accountability imperatives undermine the local level responsiveness to staff and communities needed to improve services. Secondly, greater understanding needs to be generated around the ‘how’ of LD—such that those undertaking LD, health managers and policymakers become more comfortable in working with emergence and tailoring the package of LD initiatives to the specific context. For those wishing to test or implement packages of LD, our experience suggests the need to be intentional about repeatedly engaging with (senior) managers about what a distributed relational leadership is and why it is important, advocating for visible senior support for the practice of relational leadership to generate commitment to what is ultimately a long-term process.

Our action-learning approach has implications for the interpretation of our findings. Similar to the notion of ‘embedded’ health policy and systems research (Koon *et al.* 2013), there is no separation attempted between research and LD, and we are unable to comment on how the gains from our LD interventions would have differed if they were not accompanied by research processes. Moreover, as the research processes mirrored aspects of usual LD practice, they may well have strengthened impacts. For example, the initial stakeholder engagement between senior management and LD consultants that is an important and common precursor of LD (Sharlow *et al.* 2009; Cummings *et al.* 2013) was achieved through action learning processes. Our approach also mirrored the usual LD practice of including follow-up stakeholder interviews to support and deepen learning (see e.g. Chase *et al.* 2015). A final strength of our action-learning design lies in comparison with experimental designs. Given the nature of LD—where interventions need to be emergent and tailored to context—developing an LD model and measuring its outcomes using an experimental design will not provide insights into how or why the model had impact or how it should be adjusted for different settings (Ely *et al.* 2010). In our experience, the ethos of action learning, on the other hand, is able to generate the rich, context specific lessons not only about what may be needed to strengthen leadership in similar settings but also what sort of effects the LD response might be generating.

## Conclusion

In conclusion, this experience suggests that processes of relational LD can promote the relationships necessary for effective team engagements, can encourage actors to trust each other to exercise productive discretion and can enhance the ability of managers to engage with their colleagues in a more supportive way. Nonetheless, the space and time available for relational leadership in this setting was limited by the dominance of bureaucratic management and accountability processes. Given this context, the SDMT and FMs had had limited exposure to relational leadership approaches and needed to experience them in order to understand their benefits. It is crucial that higher-level managers gain greater understanding of these problematic experiences and take them into consideration as they seek to strengthen health system governance.
